# The Intergenerational Impact of a Slow Pandemic: HIV and Children

**DOI:** 10.1002/cad.20358

**Published:** 2020-08-23

**Authors:** Geoffrey Peter Garnett

**Affiliations:** ^1^ Bill and Melinda Gates Foundation TB and HIV Program Strategy Team Seattle WA

## Abstract

Human immunodeficiency virus (HIV) has, over the last four decades, infected millions of young women and their children. Interventions developed in parallel with the spread of the virus have been able to reduce rates of vertical transmission from mother to child. The impact of HIV in children can be direct in children living with HIV (CLHIV) and exposed to HIV and uninfected, or indirect through impacts on their parents, caregivers, and family. In 2018, the United Nations joint programme on AIDS (UNAIDS) estimated that 1.7 million children were living with HIV, 160,000 were newly infected with HIV, and 100,000 died from HIV. Improvement in treatment regimens can improve the life chances of children, but adherence to treatment is a problem, especially for adolescents. Injectable long acting treatments, or interventions to improve service delivery and support for adolescents living with HIV may improve treatment success. In addition to failures of HIV prevention and treatment in CLHIV, there are concerns over exposure to the virus and antivirals leading to delayed child development. To improve the wellbeing of children affected by HIV, social support is necessary, but we need to find ways of enhancing the impact of interventions, perhaps through combining them.

Human Immunodeficiency Virus (HIV), the cause of Acquired Immune Deficiency Syndrome (AIDS) generated an unusual plague, with slow progress from infection to disease, an exceptionally high fatality rate, and patterns of transmission leading to infection and disease in young adults and their infant children. With an average duration of infectiousness of roughly a decade, HIV in the absence of treatment has generated a pandemic, an impact, and a response that has played out over four decades and will likely continue playing out for many more decades. Mainly sexually transmitted, the virus can be vertically transmitted, with severe adverse consequences, from mother to child during pregnancy and breastfeeding. In addition, morbidity and mortality among parents and caregivers, along with exposure to virus and drugs short of infection, can influence the development and wellbeing of children. We have been unable to develop a vaccine or cure for HIV, but primary prevention and antiretroviral treatment have altered the course of the pandemic and changed the effects seen among children and adolescents. Over time, questions about the direct and indirect impact of HIV on child and adolescent development have been addressed and interventions developed. Many of the worst effects of the virus have been countered, but access to interventions is heterogeneous and HIV continues to impair the wellbeing of too many children.

In this commentary, I provide a brief review of the impact of the HIV pandemic on parents, caregivers, and children, and describe the current epidemiology of HIV among children. The commentary focuses on sub‐Saharan Africa where heterosexual spread of HIV, and hence infections in reproductively aged women, has been greatest. I describe a simple categorization of the ways in which HIV can affect the health and development of children. The other papers in this issue describe pathways for influence that fall within this framework. In summarizing our response to HIV in children, I highlight some recent interventions that show promise in reducing adverse effects of the HIV pandemic in children.

## The Course of the HIV Pandemic

Widespread heterosexually acquired HIV among reproductively aged women in low income settings allowed vertical transmission of the virus in utero, intrapartum, and through breastfeeding, leading to substantial morbidity and mortality among young children. First detected among men who have sex with men (MSM) in the United States (Centers for Disease Control, 1981) the syndromes associated with HIV were soon found among heterosexuals in Central Africa (Piot et al., 1984) with viral transmission to children born to women living with HIV (Ryder et al., 1989). Vertical transmission was found to be higher, at around 40%, in low‐income settings than in high income settings where it was around 25%, with much of the difference explained by continued breastfeeding (Newell, 2001). Around half of children infected with HIV died before their second birthday, but the rate of mortality was then found to decline as a function of age, with a significant proportion of children surviving to adolescence and adulthood (Marston et al., 2011). The severity of neonatal infection, connected with the point of acquisition of the virus, created heterogeneity in the hazards of mortality which influenced the pattern of prevalent HIV infection in children as a function of age (United Nations Children's Fund, 2013). Children living with HIV infected perinatally had a median survival of 1.1 years, whereas those infected through breastfeeding has a median survival of 9.4 years (Marston et al., 2011).

HIV spread widely among heterosexuals in sub‐Saharan Africa with prevalence of infection peaking at around 30% among young women in some communities with a profound impact on mortality in women and children (Gregson et al., 2007). In addition to the direct impact of HIV on child health, the morbidity and mortality experienced by young adult parents and caregivers undermines the well‐being of children, with, for a time, households increasingly headed by grandparents or children (Barnett & Blaikie, 1992). Heterogeneity in patterns of HIV incidence and prevalence generated varied levels of social and economic impact, but HIV became a development crisis (Garnett, Grassley, & Gregson, 2001).

In some countries, a well‐organized response generated changes in risk behavior reducing the spread of HIV (Hallett et al., 2006), whilst in other countries high incidence of HIV infections and AIDS deaths in adults and children continued until effective antiretroviral treatment (ART) became available and widely used (Akullian et al., 2020). Highly effective ART dramatically improved the survival of people living with HIV and reduced the risk of infection from being transmitted from those on treatment (Cohen et al., 2011). This reduction in transmission risk was first realized in the prevention of mother to child transmission (PMTCT). Initially pregnant women were treated with a single drug to prevent vertical transmission, (Newell, 2001) then with a combination of drugs during pregnancy for improved protection of infants (Mofenson, 2008), and finally in “B+” programs, where women were treated before during and after pregnancy to protect their own health and to prevent vertical transmission (Jones et al., 2019). Additional reductions in HIV incidence in young women where HIV programs have succeeded in reducing horizontal transmission have also contributed to the reduced acquisition of HIV in children.

## Categorizing the Impact of HIV in Children

**Figure 9.1:**
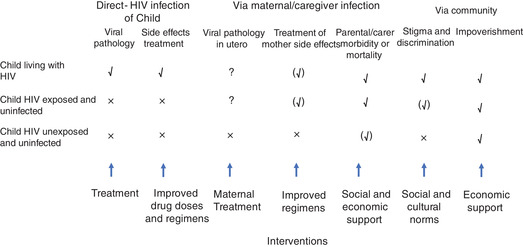
Pathways through which HIV can influence the well‐being of children and adolescents. Columns represent pathways and rows the status of children with respect to HIV. At the bottom are interventions that address the pathways. *Note*. √ represents where the effect is clearly experienced by the child in the exposure category; (√) the effect is experienced by some children in the category; (?) we are uncertain of the extent of the effect; (x) the effect is not present.

Theoretically, HIV can have direct and indirect effects on the health and development of children. Some effects are substantial and clear, whilst other effects are more subtle and their presence debatable. Children can be classified into children living with HIV (CLHIV), children exposed to HIV and uninfected (CHEU), and children unexposed to HIV and uninfected (CHUU). This last category could include those whose parent or caregiver acquired HIV after their birth. A simple classification of effects of HIV on children is provided in Figure 9.1, where the effects of virus, treatment, care, or environment can be direct in the child, via their parent or caregiver, or through their family and community. Through understanding the challenges children face we can design interventions.

Mirroring the causal pathways for harm in children interventions can be tailored to respond to the virus or treat side effects, or can be through psychological, sociological, or economic support for the child, parent, family, or community. Intervention design and adoption depends upon a good understanding of the scale of harm and the effectiveness of the intervention. When there is widespread HIV infection in children and orphan‐hood generated by adult HIV, the need for and impact of interventions is more easily measured and acted on. Over the last couple of decades infections in children and mortality in adults has been drastically reduced, so that concern has shifted to more subtle health and development effects that are both more difficult to measure and more difficult to design demonstrably effective interventions against.

## The Current Status of the HIV Pandemic Among Children

Prevention of mother to child HIV transmission (PMTCT) has greatly reduced the number of infections in children. A well‐coordinated and supported campaign, the “Global Plan toward the elimination of HIV infection among children by 2015 and keeping their mothers alive” had 10 targets, including 90% coverage of PMTCT and a reduced vertical transmission risk of 5% in breast‐feeding mothers and 2% otherwise (UNAIDS, 2011). Elimination of mother to child HIV transmission was built around four prongs: (1) To prevent HIV acquisition in women of child‐bearing age; (2) to provide access to modern contraceptives and family planning support; (3) to provide access to HIV testing and ART treatment for pregnant women; and (4) to provide HIV care and treatment to women, children and their families. By 2014, infections in children had been reduced by 48% (UNAIDS, 2015) and work continued, with improved coverage of pregnant women with ART and early infant diagnosis and treatment through the “Start Free, Stay Free, AIDS Free” framework (UNAIDS, 2019a).

The latest estimates of HIV infection in children, for 2018, were published by the United Nations joint programme on AIDS (UNAIDS) in 2019 (UNAIDS, 2019b). These estimates are based on data on prevalence of infection in women of child‐bearing age, estimates of fertility in these women, coverage of ARVs among pregnant women, and the risk of vertical transmission (Stover et al., 2019). In addition, the number of children diagnosed with HIV and on treatment can be used to inform the estimates, but there is great uncertainty around most of the estimates. UNAIDS estimates that there were 37.9 million people living with HIV globally in 2018, with 1.7 million new infections and 770,000 HIV associated deaths over the year. Of people living with HIV 1.7 million were children 15 years and younger, with 160,000 new HIV infections and 100,000 deaths in children.

Most children living with HIV are in sub‐Saharan Africa. In Southern and Eastern Africa it is estimated that 900,000 children were born to HIV infected mothers, 260,000 to HIV infected mothers in West and Central Africa, 60,000 in Asia and the Pacific, 22,000 in Latin America, 7,800 in the Caribbean and 4,800 in the Middle East and North Africa. Coverage of ARTs in pregnant women is very different between and within regions, with 92% coverage in East and Southern Africa, 59% coverage in West and Central Africa, and a low of 28% coverage in the Middle East and North Africa. The treatment coverage translates into a 9% vertical transmission risk in Southern and Eastern Africa and 22% in West and Central Africa.

## Treatment Issues Concerning Children Living With HIV

### The Limits of Treatment As Prevention in PMTCT Programs

The vertical transmission risk of 9% in East and South Africa is a reduction from 18% in 2010 and translates into 81,000 new infections. It is often questioned why transmission risk remains high despite 92% coverage of ARVs. Many of these childhood infections are acquired when a young woman herself acquires HIV infection when pregnant or when breastfeeding and is not immediately treated and virally suppressed. Many infections occur in children whose mother dropped off treatment, or started treatment late during their pregnancy, whilst around 50% occurred during breastfeeding (UNAIDS, 2019b). Breastfeeding is recommended where the risks of neo‐natal and infant mortality due to diarrhea and other infections in infants not breast fed outweighs the risks of HIV.

The ongoing transmission of HIV to infants, even with very high coverage of treatment of mothers shows the limits of a strategy focused on one “prong” of the Global Plan. The need for treatment to reduce vertical transmission is the result of the public health failure that is continued HIV acquisition in adolescent girls and young women (AGYW). Prevention of HIV in AGYW would both reduce vertical transmission risk to zero and prevent any adverse consequences among children HIV exposed and uninfected, and the social and economic consequences of morbidity and mortality in mothers.

### Treatment of Children Living With HIV

If children are infected, highly efficacious treatment is available, but is challenging and can fail if children fail to take medication, or if resistance emerges; treatments can also cause side effects (WHO, 2016). Effective, less toxic dosing of drugs in both mother and child is a goal that is supported by improved regimens, with more forgiving pharmacodynamics with better bioavailability, greater barriers to resistance, and less toxicity. In resource rich settings, it is possible to pursue individualized medicine, with viral resistance testing, precisely calibrated doses, and detailed monitoring of drug levels. In low income settings, with over‐stretched health systems, simpler algorithms are needed to direct treatment, and improved regimens are an important tool facilitating easier treatment of children. The market for HIV treatment in children is both smaller and more complex than that for adults, and children have less social and cultural capital to advocate for improved access to treatment (WHO, 2016).

Dolutegravir, an integrase inhibitor, provides an interesting case study. Dolutegravir (DTG) is replacing Efavirenz (EFV) in antiretroviral treatment guidelines for LMICs (WHO, 2019). The active pharmaceutical ingredient is cheaper, the barriers for resistance are greater, and it is less toxic. Dolutegravir in combination with tenofovir and lamivudine (TLD) is replacing tenofovir, lamivudine, and efavirenz (TLE) as a preferred treatment in adults. The recommended treatment for children is abacavir, lamivudine, and dolutegravir and for neonates zidovudine, lamivudine, and raltegravir (WHO, 2019). Initial evidence supported use in children but only 50 mg tablets for children weighing over 20 kg were available. Recently, 5 mg tablets have been approved for children, which will hopefully improve treatment options (WHO, 2020).

Dolutegravir roll out in adults was delayed due to observations of neural tube defects (NTDs) in neonates in Botswana where food folate fortification is not used. The rate observed in the Tsepamo study in Botswana was significantly higher than expected with four cases from 426 births (Zash, Makhema, & Shapiro, 2018). Subsequent expansion of monitoring of births in Botswana observed one further case in 1257 further births (Zash et al., 2019). The rate of 0.3% is still three times greater than in those not taking dolutegravir and monitoring continues. Risks of NTDs must be weighed against risks of vertical transmission of HIV, maternal mortality, and drug resistance, and a woman's right to make informed decisions has been emphasized. This highlights the challenges of introducing new drugs, the need for good post marketing surveillance to detect and understand unexpected adverse outcomes, and the potential impact on child development of antiretroviral drugs.

### Challenges of Adherence to Treatment Regimens

Adherence to HIV drugs needs to be reasonably high (around 85%) and lifelong to avoid treatment failure and the evolution of resistance. This is particularly challenging for adolescents living with HIV, who may question their infection and suffer from stigma, which taking medication may highlight. One potential solution may be long acting injectable treatment, which may facilitate observed treatment ensuring that children regularly receive necessary medication. The drugs rilpivarine and cabotegravir in a combined injection providing 1–2 months of therapy are under regulatory review and may offer a strategy to help with adherence (Orkin et al., 2020; Swindells et al., 2020).

In addition to drug regimens, the characteristics of services may help children and adolescents manage their infections. Differentiated service delivery has been promoted to provide client‐centered HIV treatment that makes adherence and retention easier for stable adult patients (Duncombe et al., 2015). The concepts have been extended to children and adolescents addressing issues for children of physical growth and dose adjustments, reliance on caregivers, their need to attend school, and for adolescents their additional push for independence, sexual and reproductive health needs, and mental health challenges. Solutions promoted include mentoring of caregivers, peer support, less frequent clinic visits for those over 2 years old, aligning visits with school vacations and out of school hours, and providing the sexual and reproductive health and mental health care needed (IAS, 2020).

Such approaches hold out hope, but the challenges are great. For example, in one recent a cohort study of approximately 1,000 adolescents in South Africa, retention on ART was only 38% (Pantelic, Casale, Cluver, Toska, & Moshabela, 2020). In a systematic review of interventions to improve adolescent ART adherence few interventions were found to be effective (Reif et al., 2020). In low‐income settings none of the three interventions targeting the individual significantly improved results. One community intervention was found to improve reported adherence, and since the review, the results of this community‐randomized trial on treatment success have been presented. The Zvandiri cluster randomized trial of community adolescent treatment supporters (CATS) supports adolescents with HIV in treatment in Zimbabwe. CATS are adolescents and young people also living with HIV who are provided training and supervision. They work through home visits, support groups, and SMS messaging, the intensity of which depends on whether the PLHIV is “stable” or not. The study randomized clinics with 213 adolescents in the intervention clinic receiving support and 287 adolescents in control clinics receiving standard of care. The researchers observed a 42% (*p* = 0.03) reduction death or failure to suppress virus in the intervention. Despite this effectiveness, 52/209 of children in the intervention arm still died or failed to suppress the virus (Mavhu et al, 2019).

## Child Development and HIV

Clearly, HIV infection leads to poor outcomes in children infected with the virus, even with effective treatment. With effective antiretrovirals survival is much better, and treatment recommendations are now that all children infected with HIV receive ARVs (WHO, 2016). Many children still die, 100,000 in 2018. For all the children that die there are more that survive, but with stunted growth and poor cognitive development caused directly by the virus or indirectly by the side effects of drugs (Wedderburn et al., 2019).

There have always been more children exposed to HIV born uninfected than infected, and the impact of HIV on these children has been a contentious issue. As evidence accrues, it appears that some of the adverse development effects seen in CHEU were found when mothers were untreated. There is still though evidence of poorer early growth a neurodevelopment in CHEU, which has been explained through the direct effect of exposure to antivirals or through an exacerbation of other risk factors associated with poor growth (Wedderburn et al., 2019). The evidence continues to be mixed (Rotheram‐Borus et al., 2019). For example, in a study of under two years‐olds there was definitive delay in development of language and motor skills in 205 CHEU (Ntozini et al., 2020).

In contrast in this issue, measures of cognitive development, psychological adjustment, and quality of life are explored as outcomes. Adverse outcomes are found for CLHIV but not for those exposed and uninfected. However, the results also show the harms of toxic stress, lifetime adversity, and lack of social support, all of which can increase in families and communities if there is a high burden of morbidity and mortality associated with HIV.

In low‐income settings, adolescents face many challenges. Orphans and vulnerable children suffer from psychological and sociological problems (Nyamukapa et al., 2010), allowing a vicious cycle, where HIV in the parent undermines the life chances of the child increasing risks of the child themselves acquiring HIV (Gregson et al., 2005). Risks of HIV are a function of the social, cultural, and economic situations young women find themselves in, often with transactional sex increasing exposure to HIV (Wamoyi, Stobeanau, Bobrova, Abramsky, & Watts, 2016). HIV is not generally a high priority, even for those who objectively have a high risk of acquisition. Young women prioritize their status and relationships and worry more about pregnancy risks (Majola, 2014).

DREAMS (Determined Resilient Empowered, AIDS‐Free, Mentored Safe) is a comprehensive PEPFAR (Presidents Emergency Plan for AIDS Relief)‐funded program for AGYW which provides social support, counseling, parental support, HIV testing and family planning (Saul et al., 2018). An ongoing evaluation should tell us how well improved economic empowerment, counseling and support have been able to reduce HIV incidence. Even if HIV incidence is not reduced endpoints such as improved economic opportunities, mental health, and reduced experience of violence may be worthwhile. Often individual interventions can have significant but small effects, but it is hoped that combining interventions leads to synergies.

An example of synergies was observed in a cohort of AGYW in South Africa. Combining safe schools, parental support, and cash transfers generated greater benefits than the interventions in isolation, reducing abuse by 51%, improving mental health by 31%, progress in schools by 27%, reducing risky sex by 17%, and decreasing experience of violence by 30% (Cluver et al., 2019).

## Conclusions

As the HIV epidemic has progressed the highest levels of child mortality and social disadvantages have been ameliorated through effective treatment and prevention. Nonetheless, too many parents and children continue to be infected with and die from HIV. For children to be exposed to or infected with HIV is a failure of public health. However, controlling HIV is a long‐term endeavor with the burden of infection and disease and the need for treatment measured in decades. Maintaining attention on HIV as new public health crises emerge will be difficult, but without attention HIV could spread widely once again, infect and expose a new generation of children and undermine their development.
